# Epileptic Seizures in Critically Ill Patients: Diagnosis, Management, and Outcomes

**DOI:** 10.3390/jcm9072218

**Published:** 2020-07-13

**Authors:** Mathilde Holleville, Gwenaëlle Jacq, François Perier, Candice Fontaine, Stephane Legriel

**Affiliations:** 1 Department of Anaesthesiology and Critical Care, Hôpitaux Universitaires Paris Nord Val de Seine, Hôpital Beaujon, 100 Boulevard du Général Leclerc, 92110 Clichy, France; mathildeholleville@gmail.com; 2 IctalGroup, 78150 Le Chesnay, France; gjacq@ch-versailles.fr (G.J.); francoisperier@hotmail.fr (F.P.); candicefontaine@yahoo.com (C.F.); 3 Intensive Care Department, Centre Hospitalier de Versailles, 177 rue de Versailles, 78150 Le Chesnay CEDEX, France; 4 Medical Intensive Care Unit, Assistance Publique-Hôpitaux de Paris, CHU Henri Mondor, 51 Avenue du Maréchal de Lattre de Tassigny, 94010 Créteil, France; 5 Medical-Surgical Intensive Care Unit, Hopital Paris Saint Joseph, 185 Rue Raymond Losserand, 75014 Paris, France; 6 UVSQ, INSERM, University Paris-Saclay, CESP, Team « PsyDev », 94800 Villejuif, France

**Keywords:** seizures, status epilepticus, critically ill, electroencephalography, adults, children

## Abstract

Epileptic seizures in critically ill patients represent a major source of concern, because they are associated with significant mortality and morbidity rates. Despite recent advances that have enabled a better understanding of the global epidemiology of this entity, epileptic seizures in critically ill patients remain associated with a high degree of uncertainty and numerous questions remain unanswered. The present Special Issue aims to invite authors to contribute original research articles as well as review articles related to all aspects of epileptic seizures in critically ill patients, diagnosis, management, and outcomes.

Epileptic seizures in critically ill patients represent a major source of concern, because they are associated with significant mortality and morbidity rates [1]. Seizures may be convulsive or nonconvulsive. They may also be prolonged in duration reaching the diagnosis criteria for status epilepticus. They can occur in various settings, and can involve a large spectrum of multidisciplinary teams, including but not limited to medical physicians and nurses from emergency departments, operating rooms, or intensive and neurocritical care units.

Recent advances have enabled a better understanding of the global epidemiology of this entity [2,3,4]. The greater implementation of continuous EEG monitoring has certainly contributed to this [5,6]. Seizures diagnostic relies on having good knowledge of the different electro-clinical presentations, themselves illustrated by the recent update in the classifications of seizures and status epilepticus [7,8]. Management of seizures associates general measures with organ failure supportive care, according to patient’s severity, antiepileptic treatment appropriate for the electrical and clinical pattern in the patient, investigations for a cause and etiological treatment, and electroencephalography monitoring [9].

However, epileptic seizures in critically ill patients remain associated with a high degree of uncertainty, and numerous questions remain unanswered [10]. Indeed, further progress is necessary regarding diagnostic procedures to better identify situations in which electroencephalogram should be performed in emergency situations. While the contribution of continuous electroencephalographic monitoring is intuitively obvious, we should also progress in demonstrating its superiority when compared to the sequential approach [11]. Additional questions should be addressed, like who must do it [12,13,14], with which equipment and technics types [15,16], using which electroencephalographic montages [17], for how long, within which emergency time frame [14], and ultimately for which specific patient population [18]?

Beyond the unspecific management description, we do not really know who to treat and when to treat them with regard to epileptic seizures in critically ill patients, nor what the most efficient treatments are [19,20,21]. The role of general anesthesia and which agents should be used remains debated, just like the place of adjuvant anticonvulsant and neuroprotective strategies [22,23,24]. Furthermore, additional progress needs to be made to define the roles and interactions between the protagonists involved in the management of these patients: nurses, emergency physicians, intensivists, neurointensivists, neurologists, and neurophysiologists [25,26].

Finally, few data are available regarding the large spectrum of presentation, the underlying conditions, the immediate severity, and their association with outcomes in critically ill patients with epileptic seizures in each of the various settings cited above.

The present Special Issue invites authors to contribute original research articles, as well as review articles related to all aspects of epileptic seizures in critically ill patients, diagnosis, management, and outcomes.
